# Chromosome Level Genome Assembly and Annotation of Highly Invasive Japanese Stiltgrass (*Microstegium vimineum*)

**DOI:** 10.1093/gbe/evab238

**Published:** 2021-10-28

**Authors:** Dhanushya Ramachandran, Cynthia D Huebner, Mark Daly, Jasmine Haimovitz, Thomas Swale, Craig F Barrett

**Affiliations:** 1 Department of Biology, West Virginia University, USA; 2 USDA Forest Service, Northern Research Station, Morgantown, West Virginia, USA; 3 Dovetail Genomics, LLC, Scotts Valley, California, USA

**Keywords:** long read sequencing, polyploidy, transposable elements, rapid adaptation, invasion genomics, Poaceae, genome evolution

## Abstract

The invasive Japanese stiltgrass (*Microstegium vimineum*) affects a wide range of ecosystems and threatens biodiversity across the eastern USA. However, the mechanisms underlying rapid adaptation, plasticity, and epigenetics in the invasive range are largely unknown. We present a chromosome-level assembly for *M. vimineum* to investigate genome dynamics, evolution, adaptation, and the genomics of phenotypic plasticity. We generated a 1.12-Gb genome with scaffold N50 length of 53.44 Mb respectively, taking a de novo assembly approach that combined PacBio and Dovetail Genomics Omni-C sequencing. The assembly contains 23 pseudochromosomes, representing 99.96% of the genome. BUSCO assessment indicated that 80.3% of Poales gene groups are present in the assembly. The genome is predicted to contain 39,604 protein-coding genes, of which 26,288 are functionally annotated. Furthermore, 66.68% of the genome is repetitive, of which unclassified (35.63%) and long-terminal repeat (LTR) retrotransposons (26.90%) are predominant. Similar to other grasses, *Gypsy* (41.07%) and *Copia* (32%) are the most abundant LTR-retrotransposon families. The majority of LTR-retrotransposons are derived from a significant expansion in the past 1–2 Myr, suggesting the presence of relatively young LTR-retrotransposon lineages. We find corroborating evidence from Ks plots for a stiltgrass-specific duplication event, distinct from the more ancient grass-specific duplication event. The assembly and annotation of *M. vimineum* will serve as an essential genomic resource facilitating studies of the invasion process, the history and consequences of polyploidy in grasses, and provides a crucial tool for natural resource managers.

**Significance**

The current lack of genomic resources for the invasive Japanese stiltgrass—and thousands of other invasive species globally—severely limits our understanding of the invasion process and hinders decision-making for effective management and control. In this study, we present a chromosome-level genome assembly and annotation of Japanese stiltgrass, a problematic weed in eastern North America, identifying a clear history of polyploidy and recent activity of transposable elements. The ultimate goal is to advance genomic studies to better understand the dynamics of nonnative species during the various invasion phases, thereby providing insights into effective control strategies to manage current and future invasions.

## Introduction

Invasive species cause billions of dollars in damage annually, and are considered the second greatest threat to native biodiversity after habitat loss (Pejchar and Mooney 2009; Simberloff 2013). Yet, genomic resources for invasive species are generally lacking relative to other economically important species such as crops, microbial pathogens, and many animal systems (McCartney et al. 2019). Almost half of the native species in the United States are at risk of extinction either due the direct effects of introduced species or impacts combined with other processes (Pimentel et al. 2005). Efforts to identify and eradicate newly introduced species are hampered by the lack of resources needed to predict how and why some species will become invasive. Genomics has become an increasingly valuable and cost-efficient tool to predict and diagnose invasions (Chown et al. 2015; Hamelin and Roe 2020). Genomics can provide novel insights on the roles of genetic variation, multiple introductions, admixture, introgression, and rapid adaptation (Schrader et al. 2014; Kreiner et al. 2019; Bertolotti et al. 2020; Olazcuaga et al. 2020; Yainna et al. 2020; Malinsky et al. 2021). For instance, a high-quality genome is useful for genome-wide scans of selection, trait association mapping, and timing invasion events (Nielsen et al. 2005; DeGiorgio et al. 2016; North et al. 2021). With improved understanding and forecasting at each stage of the invasion process, managers can make decisions on invasions much more accurately than in the past (Bergeron et al. 2019; Keriö et al. 2020). Hence, sequencing whole genomes for these nonmodel organisms provide crucial tools to efficiently manage and predict future invasions.

Japanese stiltgrass (*Microstegium vimineum*) is a shade-tolerant, annual, C4 grass introduced to the eastern USA from Asia in the early 1900s that has spread to 30 US states and Canada. This species invades a range of habitats in the United States, displays a high degree of phenotypic plasticity, has a mixed mating system (outcrossing and self-fertilization), and exhibits prolific reproductive output with seeds being viable in the soil up to 5 years (Barden 1987; Redman 1995; Gibson et al. 2002; Nees 2016; Culpepper et al. 2018). Considerable research interest has been focused on unraveling potential links between ploidy levels and invasiveness, as most invasive plant species are polyploids (Pandit et al. 2011; te Beest et al. 2012). Japanese stiltgrass is an ideal system for the study of rapid adaptation of invasive species, being a putative polyploid in addition to the aforementioned features (2*N* = 20 as opposed to the “base” 2*N* = 10 among members of Andropogoneae; Watson and Dallwitz 1992).

Here, we present a high-quality, chromosome-level assembly, and annotation for *M. vimineum*, by integrating PacBio sequencing, Omni-C scaffolding, and RNAseq. The genome will lay groundwork for further investigation of traits allowing *M. vimineum* to adapt and thrive as an invasive species. Further, this genome will provide an important genomic resource for studies of rapid adaptation in invasive plants, help elucidate the history and consequences of polyploidy in grasses, and provide a tool for natural resource scientists and managers.

## Results and Discussion

### Genome Sequencing and Assembly

**Fig. 1.**
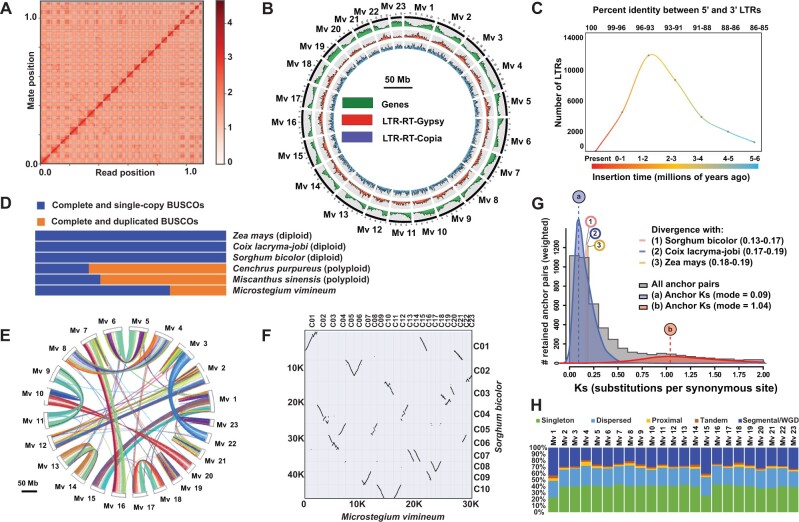
(*A*) Linkage density heatmap of the *Microstegium vimineum* genome. The *x* and *y* axes represent the mapping positions of the first and second read in a read pair, respectively. The diagonal lines from lower left to upper right in the plot represent each of the 23 *M. vimineum* pseudochromosomes. Dots (sequences) outside the diagonal are likely repetitive sequences that occur in multiple chromosomes. (*B*) Circos plot of *M. vimineum* genome assembly showing distributions of genes (green), *Gypsy* LTR-RTs (red), and *Copia* LTR-RTs (blue). (*C*) Insertion age estimates of LTR-retrotransposons in Ma based on a grass-specific LTR mutation rate (Ma and Bennetzen 2004). (*D*) BUSCO assessment results of orthologs among *M. vimineum*, closely related diploids (*Sorghum bicolor*, *Coix lacryma-jobi*, *Zea mays*), and polyploids (*Miscanthus sinensis* and *Cenchrus purpureus)*. (*E*) Interchromosomal synteny with links representing syntenic blocks between *M. vimineum* chromosomes. (*F*) Macrosynteny dotplot of *M. vimineum* and *S. bicolor* chromosomes displaying large-scale duplications, inversions, and translocations. (*G*) The frequency distributions of synonymous substitution rates (Ks) of homologous gene pairs located in the collinearity blocks of *M. vimineum*. The Ks distribution for *M. vimineum* is shown in gray, with two WGD peaks indicated in blue and red. The vertical lines labeled “a” and “b” indicate the modes of these peaks, which are taken as Ks-based WGD age estimates. The numbered vertical lines represent rate-adjusted mode estimates of one-to-one ortholog Ks distributions between *M. vimineum* and closely related species, representing speciation events. (*H*) Distributions of gene duplicate origins across each chromosome in *M. vimineum* genome.

We generated a high-quality, chromosome-level genome assembly of *M. vimineum* using PacBio and Dovetail Omni-C libraries. Using approximately 60 Gb of PacBio long read data, we initially assembled 5,261 de novo contigs with N50 of 605 kb. In parallel, a total of 73.21 Gb (30× coverage) of short read sequence data were produced by Illumina HiSeqX from Dovetail’s Omni-C libraries to achieve chromosome-scale scaffolding. The initial assembly was significantly improved with Omni-C data using the HiRise pipeline (fig. 1*A*), which produced a final assembly consisting of 462 scaffolds spanning 1.1 Gb in length, with the scaffold N50 size of 53 Mb (table 1). The final assembly covers 99.96% of 1.3 Gb genome size and interestingly, about 99.11% of assembled genome were anchored into 23 pseudochromosomes (size range 20.9–68.32 Mb), corresponding closely to the expected number of 20 chromosomes (fig. 1*A*).

**Table 1:** Summary of the Genome Assembly and Annotation

Genome assembly	Estimated genome size	1.2 Gb
	N50 scaffold length	53.04 Mb
	L50	10
	N90 scaffold length	33.01 Mb
	L90	20
	Longest scaffold	68.32 Mb
	No. of scaffolds	463
BUSCO	Complete	3930 (80.2%)
	Duplicate	1159
	Fragmented	108
	Missing	859
	Total BUSCO groups searched	489
Transposable elements	LTR-retrotransposons	25.77%
	LINEs	1.13%
	DNA-transposons	3.96%
	Rolling circles	0.13%
	Unclassified/unknown	35.63%
	Total	66.48%
Protein-coding genes	No. of gene models	39,604
	Functionally annotated	26,288
	Mean gene length	1,394 bp
	Mean no. of exons per gene	5
	Mean exon length	256 bp
	Mean intron length	679 bp

### Repeat and Gene Annotation

Over half of the genome is composed of repetitive elements (66.68%, 745.92 Mb; table 1). Class I long-terminal repeat (LTR) retrotransposons are predominant, constituting 39.08% of the assembled genome. Similar to other grasses, the most abundant LTR-retrotransposon family present in *M. vimineum* genome is *Gypsy* (41.07%), followed by *Copia* (32%) (Baucom et al. 2009; Paterson et al. 2009; Schnable et al. 2009; Tian et al. 2009). *Gypsy* elements are distributed in gene-poor regions in most pseudochromosomes, whereas *Copia* shows a more even distribution (fig. 1*B*). Calibrated sequence divergence of 5′- and 3′-terminal repeats revealed that most LTR-retrotransposons insertions appear to have occurred 1–2 Ma (fig. 1*C*), suggesting recent activity of LTR-retrotransposons and preponderance of young LTR lineages in the genome.

We predicted 39,604 genes spanning 55.22 Mb (approximately 4.9%) of the genome, with an average gene length of 1,394 bp (table 1). A total of 26,230 genes were functionally annotated. We evaluated the completeness of the predicted gene sets and extent of gene duplication with 4,896 BUSCOs from the Poales database (v10; Manni et al. 2021), of which 3,930 (80.2%) were complete, indicating a relatively complete genome assembly and gene prediction (table 1). An interesting observation among the complete BUSCO’s was the presence of 1,159 (30%) complete duplicated copies. This degree of duplication is comparable with, but lower than that seen in the polyploids *Miscanthus sinensis* (Mitros et al. 2020) and *Cenchrus purpureus* (Yan et al. 2021) (fig. 1*D*).

### Whole-Genome Duplication in *M. vimineum*

Syntenic blocks in *M. vimineum* are displayed in figure 1*E*. Investigation of collinear orthologs between *M. vimineum* and the diploid *Sorghum bicolor* revealed a 2:1 (*M. vimineum:S. bicolor*) synteny pattern with evidence of duplications, translocations, and inversions confirming the occurrence of whole-genome duplication (WGD) in *M. vimineum*. Chromosomes 13 and 14 in *M. vimineum* are apparent homeologs, displaying collinearity along their entire length to *Sorghum* chromosome 7. Large-scale inversions are observed on *M. vimineum* chromosomes 5 and 6, which are syntenic to *Sorghum* chromosome 2. Inverted homeologs within chromosomes 17 and 18 of *M. vimineum* display clear collinearity to *Sorghum* chromosome 8. Chromosomes 4 and 8 of *M. vimineum* are syntenic to *Sorghum* chromosome 5, but with two large-scale inversions in *M. vimineum* chromosome 8 (fig. 1*F*).

The Ks peaks in figure 1*G* indicate two WGD events: 1) a paleoduplication event shared by all grasses at Ks = 1.04, estimated at 80–90 Ma (Paterson et al. 2004), and 2), and a *M. vimineum*-specific WGD at Ks = 0.09. The majority of duplicates in *M. vimineum* were derived from WGD/segmental (28.5%) and dispersed (27.5%) duplications, corroborating polyploidization followed by considerable chromosomal reshuffling in *M. vimineum* (fig. 1*H*). At a minimum, this suggests the *M. vimineum*-specific duplication likely occurred in the last ∼10 Ma, but additional taxon sampling is needed to more accurately estimate the timing of this event.

## Conclusion

We generated a high-quality, chromosome-scale genome assembly, and annotation for *M. vimineum* using PacBio sequencing and Omni-C technology. Genome quality assessment indicated a highly contiguous, accurate assembly and annotation, revealing recent WGD and transposon activity. Given the paucity of sequenced genomes for invasive species, this genome will serve as an important resource to study invasive species at the genomic level. Due to the varying abilities of introduced species to establish in a new environment, decision-making regarding resource allocation, mitigation, and management has always been uncertain; availability of genomic information for nonnative species may provide new solutions (Hamelin and Roe 2020). Whole-genome information expedites downstream population genomic studies on the role of multiple introductions, admixture, and adaptive ramifications of novel genotypes allowing “exploration” of novel phenotypic space, phenologies, and ecological interactions (Bertolotti et al. 2020). In addition, this genome will facilitate studies on the role of epigenetic variation and mobile elements of the genome to delineate their roles in rapid adaptation to the introduced range. These latter processes may allow novel phenotypes and gene expression modifications against the predicted genomic background of low allelic diversity in many invasive species (Mérel et al. 2021). Further, comparative genomics and evolutionary studies of invasive versus noninvasive grasses or other plants, animals, and microbes may help to identify genomic commonalities characteristic of successful invaders.

## Materials and Methods

### Sample Collection and DNA Extraction

Florets containing seeds were collected and mixed from three populations in the Potomac Ranger District (PRD) of the Monongahela National Forest (MNF) near Petersburg, WV, and three populations in the Cheat Ranger District (CRD) of the same forest near Parsons, WV. Florets were air-dried for 3 months, and cold–dry stratified at 4 °C for 1 year. One plant was also grown from seed-bank soil collected along the Monongahela River Rail Trail (RT) in Morgantown, WV. Seeds were germinated over 2 weeks in a Conviron growth chamber under temperatures of 25 °C/15 °C (12-h day/12-h night), approximately 70% humidity, and 500 µmol m^−2^ s^−1^ light. RT seedlings were transplanted into potting soil. After germination, day length was increased to 14 h and night temperature was increased to 20 °C. The complete shoot of one individual was harvested from each location (PRD, CRD, and RT). Twenty-five grams of fresh, young, green leaf tissue from one PRD accession was chosen for genome sequencing; tissue was flash-frozen in liquid nitrogen and stored at −80 °C for 1 month before shipping on dry ice. The remainder of these individuals were stored at −80 °C upon flowering with a voucher specimen of each deposited at the Northern Research Station, USDA Forest Service Herbarium. Further, tissue was harvested from these frozen samples for RNA-seq analysis. Approximately 0.2 g of tissue was harvested from you, developing tissues for: 1) leaves, 2) roots, 3) cleistogamous inflorescences (covered by leaf sheaths at the nodes), and 4) apical, chasmogamous inflorescences. Tissues were flash frozen as above, stored at −80 °C, and shipped on dry ice to GeneWiz, Inc. (South Plainfield, NJ) for RNA sequencing.

### PacBio Library Sequencing

Total genomic DNAs were extracted from leaf tissues to construct sequencing libraries (see Supplementary Material online). PacBio SMRTbell libraries (∼20 kb) were constructed using the SMRTbell Express Template Prep Kit 2.0 (PacBio, Menlo Park, CA), following the manufacturer’s protocol. Libraries were bound to polymerase using the Sequel II Binding Kit 2.0 (PacBio) and loaded onto a PacBio Sequel II at Dovetail Genomics, LLC. Sequencing was performed on two PacBio Sequel II 8M SMRT cells. PacBio reads were assembled using the Wtdbg2 pipeline (Ruan and Li 2020). Contaminants and “haplotigs” (contigs from a single, alternative haplotype) were filtered using Blobtools v1.1.1 (Laetsch and Blaxter 2017) and purge_dups v1.1.2 (Guan et al. 2020; see Supplementary Material online).

### Dovetail Omni-C Library Preparation and Sequencing

For Dovetail Omni-C libraries, chromatin was fixed with formaldehyde, extracted, and randomly digested with DNAse I. Chromatin ends were repaired and ligated to a biotinylated bridge adapter, followed by proximity-ligation of adapter-containing ends. After proximity ligation, crosslinks were reversed and DNA was purified. Purified DNA was treated to remove biotin that was not internal to ligated fragments, and sequencing libraries were generated using NEBNext Ultra enzymes and Illumina-compatible adapters. Biotin-containing fragments were isolated using streptavidin beads before PCR enrichment of each library. The library was sequenced on an Illumina HiSeqX platform to produce approximately 30× sequence coverage depth. HiRise was used for scaffolding, a pipeline designed specifically for proximity ligation data (Putnam et al. 2016), requiring mapping quality >50 reads. Dovetail OmniC library sequences were aligned to the draft input assembly using bwa (version 0.7.17; https://github.com/lh3/bwa; Li and Durbin 2009). Separations of OmniC read pairs mapped within draft scaffolds were analyzed by HiRise to produce a likelihood model for genomic distance between read pairs, and used to identify and break putative mis-joins, to score and make prospective joins.

### RNA-Seq

Total RNAs were extracted using the QIAGEN RNeasy Plus Kit following manufacturer protocols. Total RNAs were quantified using the Qubit RNA Assay and a TapeStation 4200. Prior to library preparation, DNase treatment was performed followed by AMPure (Beckman Coulter Life sciences) bead cleanup and QIAGEN FastSelect HMR rRNA (QIAGEN) depletion. Libraries were prepared with the NEBNext Ultra II RNA Library Prep Kit following manufacturer protocols and run on an Illumina NovaSeq6000 in 2 × 150 bp configuration.

### Assessment of Genome Assembly Quality

Completeness of the genome and predicted gene quality was assessed using Benchmarking Universal Single-Copy Orthologs (BUSCO v3.0.1; Simão et al. 2015). The poales_odb10 lineage-specific profile that contains 4,896 BUSCO gene groups was evaluated against our chromosome-level assembly.

### Gene Prediction and Annotation

Coding sequences from *Coix lacryma-jobi* (PRJNA544872)*, Miscanthus sacchariflorus* (PRJNA435476)*, Saccharum* “hybrid cultivar” (PRJNA272769), *S. bicolor* (PRJNA331825), and *Zea mays* (PRJNA10769) were used to train the ab initio model for *M. vimineum* using AUGUSTUS (version 2.5.5; Stanke et al. 2008). The same coding sequences were also used to train a separate ab initio model for *M. vimineum* using SNAP (v2006-07-28; Korf 2004). RNA-seq reads were mapped to the genome using STAR (v2.7; Dobin et al. 2013) and intron–exon boundary hints were generated. AUGUSTUS was then used to predict genes in the repeat-masked reference genome. Only genes predicted by both SNAP and AUGUSTUS were retained in the final gene sets. Genes were further characterized for putative functions by performing a BLAST search of peptide sequences against the UniProt database (UniProt Consortium 2021). tRNAs were predicted using the software tRNAscan-SE (version 2.05, Chan and Lowe 2019).

### Repeat Analysis

Repeat families in *M. vimineum* were identified de novo and classified using RepeatModeler (version 2.0.1; www.repeatmasker.org/RepeatModeler; Flynn et al. 2020) and EDTA v1.9.4 (Ou et al. 2019). RepeatModeler uses RECON (version 1.08; Bao and Eddy 2002) and RepeatScout (version 1.0.6; Price et al. 2005) for de novo identification. Class I LTR-retrotransposons (LTR-RT) were further predicted and annotated using RepeatModeler and EDTA. Both tools use a series of LTR-RT identification programs such as LTR-harvest, LTR-finder, and LTR-retriever. Redundant and nested insertions were removed by EDTA. Intact LTR-RTs were identified and approximate insertion times (Ma) were estimated using LTR-retriever (based on a grass-specific LTR substitution rate of 1.3 × 10^−8^ mutations per site per year; Ma and Bennetzen 2004). EDTA further uses TIR-learner and Helitron-scanner to predict and annotate Class II DNA transposons and helitrons, or rolling circle DNA transposons (Feschotte and Wessler 2001; Kapitonov and Jurka 2001). The custom repeat library obtained from RepeatModeler and EDTA was used to discover, identify, and mask repeats in the assembly using RepeatMasker (version 4.1.0; http://www.repeatmasker.org;Smit et al. 2013).

### Detection of WGD Events

To investigate WGD events in *M. vimineum* genome, the distribution of synonymous substitution (Ks) rates was obtained from protein-coding sequences and compared with closely related grasses, for example, *S. bicolor*, *Coix lacryma-jobi*, and *Z. mays.* Paralog and ortholog pairs were detected from protein sequence data and the associated Ks values were calculated using the tool “ksrates” (https://github.com/VIB-PSB/ksrates;Sensalari et al. 2021). A mixed Ks plot was generated by comparing ortholog-Ks estimates to the paralog-Ks scale of *M. vimineum*. MCScan (https://github.com/tanghaibao/jcvi/wiki/MCscan-(Python-version); Tang et al. 2008) was used for pairwise synteny (protein) search with the LSAT results of *M. vimineum* versus *S. bicolor*. The MCScan “jcvi.graphics.dotplot” module was used to visualize pairwise synteny results. Further, the genes of *M. vimineum* genome were classified into singletons, dispersed, tandem, proximal, and WGD/segmental duplicates using “duplicate_gene_classifier” module within the MCScan_X tool (Wang et al. 2012), by parsing the all_Vs_all BlastP results. 

## Supplementary Material

Supplementary data are available at *Genome Biology and Evolution* online.

## Supplementary Material

evab238_Supplementary_Data
