# Supplementary material for: Chromosome Level Genome Assembly and Annotation of Highly Invasive Japanese Stiltgrass (Microstegium vimineum)
Source: Genome Biol Evol. 2021 Oct 28;13(11):evab238. doi: 10.1093/gbe/evab238 (PMC8598173; doi:10.1093/gbe/evab238)
Supplement: evab238_Supplementary_Data [file evab238_supplementary_data.docx]

**Supplementary Material**

**DNA extraction for PacBio Library preparation**

Total genomic DNAs were extracted using the CTAB method (Doyle and Doyle, 1987). DNA concentration was quantified with a Qubit Fluorometer v2.0 using the dsDNA Broad Range assay (Thermo Fisher Scientific, Waltham, Massachusetts, USA), yielding a total of 11.2µg. Genomic DNA was further purified using a Qiagen Mini Column, following the manufacturer’s protocol (Qiagen, Hilden, Germany). The final DNA sample was resuspended in 50µl of Tris-EDTA Buffer (pH 8.0). The sample was quantified and checked for high molecular weight via Qubit, NanoDrop (Thermo Fisher Scientific, Waltham, Massachusetts, USA), Pulse Field Gel Electrophoresis, and an Agilent TapeStation 4200 (Santa Clara, California, USA).

**PacBio reads assembly using Wtdbg2 Assembler and Scaffolding with Dovetail Omni-C**

Wtdbg2 (Ruan and Li, 2020) was run with the following parameters: -x sq -g 1g -L 5000. Blobtools v1.1.1 (Laetsch and Blaxter, 2017) was used to identify potential contamination in the assembly based on blast (v2.9) results of the assembly against the NT database. A fraction of the scaffolds was identified as contaminant and were removed from the assembly. The filtered assembly (filtered.asm.cns.fa) was then used as an input to purge_dups v1.1.2 (Guan et al. 2019) and potential haplotypic duplications were removed from the assembly, resulting in the final purged.fa assembly. Contig-contig read pair linkages are analyzed to produce a 3D model of the genome. This model accounts for the distance between contigs and number of supporting Omni-C read pairs between each contig. The HiRise software was then used ranks contigs based on these linkages and scaffolds were scored based on observed insertion size and evidence inside these insertion size windows (Putnam et al, 2016).

**Gene prediction and annotation**

Coding sequences from *Coix lacryma-jobi* (PRJNA544872)*, Miscanthus sacchariflorus* (PRJNA435476)*, Saccharum* ‘hybrid cultivar’ (PRJNA272769), *Sorghum bicolor* (PRJNA331825)*,* and *Zea mays* (PRJNA10769) were used to train the ab initio model for *Microstegium vimineum* using AUGUSTUS (version 2.5.5; Stanke et al. 2008). Six rounds of prediction optimization were done with AUGUSTUS. The same coding sequences were also used to train a separate ab initio model for *Microstegium vimineum* using SNAP (v2006-07-28; Korf, 2004). RNA-seq reads were mapped to the genome using STAR (v2.7; Dobin et al. 2013) and intron hits generated with the ‘bam2hints’ tools within AUGUSTUS. MAKER, SNAP and AUGUSTUS (with intron-exon boundary hints provided from RNA-Seq) were then used to predict for genes in the repeat-masked reference genome. To help guide the prediction process, Swiss-Prot peptide sequences from the UniProt database (The UniProt Consortium, Nucleic Acids Research, 2021) were downloaded and used in conjunction with the protein sequences from the species above to generate peptide evidence in the MAKER pipeline (Cantarel et al. 2008). Only genes that were predicted by both SNAP and AUGUSTUS were retained in the final gene sets. To help assess the quality of the gene prediction, AED scores were generated for each of the predicted genes in MAKER. Genes were further characterized for putative functions by performing a BLAST search of peptide sequences against the UniProt database. tRNAs were predicted using the software tRNAscan-SE (version 2.05, Chan & Lowe 2019)**.**

**Literature Cited**

Cantarel BL et al. 2008. MAKER: an easy-to-use annotation pipeline designed for emerging model organism genomes. Genome Res. 18:188–196. doi: [10.1101/gr.6743907](https://doi.org/10.1101/gr.6743907).

Chan PP, Lowe TM. 2019. tRNAscan-SE: Searching for tRNA genes in genomic sequences. Methods Mol Biol. 1962:1–14. doi: [10.1007/978-1-4939-9173-0_1](https://doi.org/10.1007/978-1-4939-9173-0_1)

Dobin A et al. 2013. STAR: ultrafast universal RNA-seq aligner. Bioinformatics. 29:15–21. doi: [10.1093/bioinformatics/bts635](https://doi.org/10.1093/bioinformatics/bts635).

Doyle, J.J.; Doyle, J.L. 1987. A rapid DNA isolation procedure for small quantities of fresh leaf tissue. Phytochemical Bulletin, v.19, p.11-15.

Guan D et al. 2020. Identifying and removing haplotypic duplication in primary genome assemblies. Bioinformatics. 36:2896–2898. doi: [10.1093/bioinformatics/btaa025](https://doi.org/10.1093/bioinformatics/btaa025).

Laetsch DR, Blaxter ML. 2017. BlobTools: Interrogation of genome assemblies. F1000Research. 6:1287. doi: [10.12688/f1000research.12232.1](https://doi.org/10.12688/f1000research.12232.1).

Putnam NH et al. 2016. Chromosome-scale shotgun assembly using an in vitro method for long-range linkage. Genome Res. 26:342–350. doi: [10.1101/gr.193474.115](https://doi.org/10.1101/gr.193474.115).

Stanke M, Diekhans M, Baertsch R, Haussler D. 2008. Using native and syntenically mapped cDNA alignments to improve de novo gene finding. Bioinformatics. 24:637–644. doi: [10.1093/bioinformatics/btn013](https://doi.org/10.1093/bioinformatics/btn013).
